# Clear cell stromal tumor of the lung with multinucleated giant cells: a report of a case with YAP1-TFE3 fusion

**DOI:** 10.1186/s13000-023-01304-0

**Published:** 2023-01-27

**Authors:** Radek Jakša, Ivana Stružinská, Michaela Kendall Bártů, Stanislav Trča, Radoslav Matěj, Pavel Dundr

**Affiliations:** 1 Department of Pathology, First Faculty of Medicine, Charles University and General University Hospital in Prague, Studničkova 2, 128 00 Prague 2, Czech Republic; 2 Department of Abdominal, Thoracic Surgery and Traumatology, First Faculty of Medicine, Charles University and General University Hospital in Prague, U Nemocnice 2, 128 00 Prague 2, Czech Republic; 3 Department of Pathology, Charles University, 3rd Faculty of Medicine, University Hospital Kralovske Vinohrady, Prague, Czech Republic; 4 Department of Pathology and Molecular Medicine, Third Faculty of Medicine, Charles University, Thomayer Hospital, Prague, Czech Republic

**Keywords:** Clear cell (hemangioblastoma-like) stromal tumor, Lung, *YAP1-TFE3 fusion*

## Abstract

**Background:**

Clear cell (hemangioblastoma-like) stromal tumor of the lung (CCSTL) is a rare pulmonary neoplasm. Recently, 9 cases of CCSTL harboring the *YAP1-TFE3* gene fusion have been described, and it has been suggested that this aberration could be a characteristic feature of this tumor.

**Case presentation:**

We here report another case of CCSTL in a 57-year-old male, which presented as a solitary lung nodule 45 mm in the greatest dimension. Microscopically, the tumor consisted of epithelioid to spindled cells with mild-to-moderate nuclear atypia, finely granular or vesicular chromatin, and small nucleoli. Nuclear indentations were a common finding. There were up to 3 mitoses per 10 HPF. The cytoplasm was slightly eosinophilic or clear. Scattered non-tumor large multinucleated cells were present. Immunohistochemically, the tumor cells showed diffuse positivity for TFE3, CD10, vimentin, and IFITM1. Other markers examined were negative, and the expression of lineage-specific markers was not found. NGS analysis revealed a fusion transcript of the *YAP1* and *TFE3* genes, and a pathogenic variant of the *MUTYH* gene.

**Conclusion:**

Our finding supports the recent data suggesting that CCSTL represents a distinct entity characterized by the recurrent *YAP1-TFE3* fusion.

**Supplementary Information:**

The online version contains supplementary material available at 10.1186/s13000-023-01304-0.

## Background

Clear cell (hemangioblastoma-like) stromal tumor (CCSTL) of the lung is a rare entity originally described by Falconieri et al. in 2013 [1]. The tumor resembles hemangioblastoma, based on some morphological features, but its immunohistochemical profile is different and the precise histogenesis of the lesion is unknown. To date, 18 cases of CCSTL have been described in the literature [1–6]. In 10 of these cases molecular analysis was performed and 9 of them showed recurrent *YAP1-TFE3* fusion. We here report another case of molecularly confirmed CCSTL with *YAP1-TFE3* fusion and discuss its clinicopathological features.

## Case presentation

### Clinical findings

A 57-year-old male was referred to the department of pneumology of our institution following the identification of a tumor located in the upper right lung lobe in January 2021. The tumor represented an incidental finding during a routine chest X-ray performed prior to inguinal hernia surgery. The subsequent PET CT confirmed a tumor mass 50 mm in the largest dimension and the patient underwent a bronchoscopic biopsy in March 2021, during which no tumor tissue was obtained. The multidisciplinary team scheduled the patient for an upper right lobe resection, which was performed in May 2021. After 12 months of follow-up, the patient is alive with no evidence of disease.

### Pathological findings

The resection specimen consisted of upper right lung lobe 160 × 135 × 40 mm and 6 lymph nodes 5—12 mm in the largest dimension. In the lung parenchyma there was a well-demarcated, nonencapsulated solid oval-shaped soft tumor 45 mm in diameter, with no connection to the bronchus. The tumor was located 2 mm beneath the pleura, which was intact. The surgical margin was free of the tumor.

**Fig. 1:**
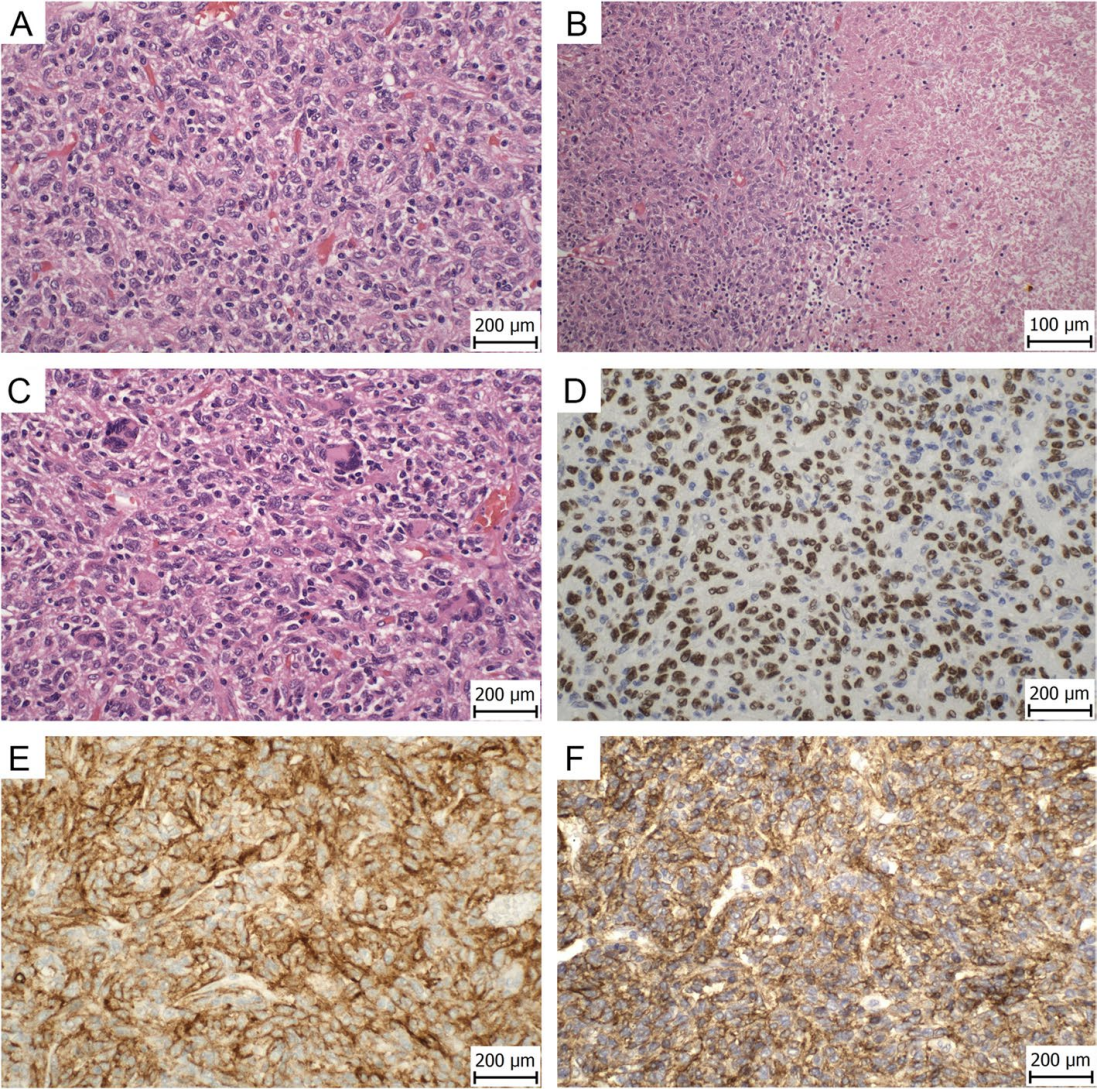
Hematoxylin–eosin staining showing tumor consisted of epithelioid to spindled cells with mild-to-moderate nuclear atypia, finely granular or vesicular chromatin, and small nucleoli (400x) **A**. In the tumor there were small foci of necrosis (200x) **B**. High-power view showed scattered large multinucleated giant cells (400x) **C**. Immunohistochemically, the tumor showed diffuse expression of TFE3 **D**, CD10 (**E**), and IFITM1 (**F**) (400x)

Microscopically, the tumor consisted of epithelioid to spindled cells with mild-to-moderate nuclear atypia, finely granular or vesicular chromatin, and small nucleoli (Fig. 1A). Nuclear indentations were a common finding. There were up to 3 mitoses per 10 HPF. The cytoplasm was slightly eosinophilic or focally clear. Scattered large multinucleated cells were present, some of them with osteoclast-like features (Fig. 1C). These cells represent a non-tumor component with immunohistochemically different profile compared to the tumor cells. Small foci of necrosis were rarely found (Fig. 1B). In the stroma, there were small foci of lymphocytes, cholesterol crystal clefts, and rare microcalcifications. There was no lymphovascular and perineural invasion. The lymph nodes were without metastases.

### Immunohistochemical finding

The immunohistochemical (IHC) analysis was performed using 4 μm thick sections of formalin-fixed and paraffin-embedded (FFPE) tissue. The list of antibodies used, including their clones, manufacturers, dilution, and staining instruments is summarized in the Supplementary table 1. The tumor cells showed diffuse positivity for CD10, IFITM1, CD99 and TFE3 (strong nuclear) (Figs. 1D-F). Focal positivity was present for antibodies against calponin and h-caldesmon. Other markers examined including cytokeratin AE1/3, TTF-1, p40, INSM1, chromogranin, synaptofysin, CD45, melan A, HMB-45, ALK, desmin, NKX2.2, FLI-1, and PAX8 were negative. The Ki-67 index (MIB1 antibody) was about 5%. The multinucleated giant cells were positive for CD163, CD68 (clone PGM1), and CD68 (clone KP1). TFE3 was negative in these cells.

### Molecular findings

**Fig. 2:**
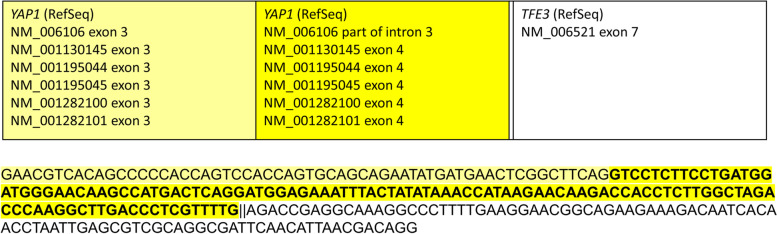
Breakpoit analysis of the *YAP1-TFE3* fusion RNA sequencing analysis revealed 265 bases long sequence (identified in 242 × reads; a copy of the fusion sequence is provided in the lower part of the figure). Verified breakpoints are indicated by *double line* and correspond to *YAP1* breakpoint (GRCh37) chr11:102,056,862 and *TFE3* breakpoint (GRCh37) chrX:48,891,297. According to the main RefSeq reference sequence NM_006106, the final fusion transcript includes exonisation of a part of *YAP1* intron 3, which corresponds to *YAP1* exon 4 according to other alternative transcripts (e.g. NM_001130145), followed by exon 7 of *TFE3* (NM_006521). The upper part of the figure shows a diagram of the sequence identification

Targeted DNA NGS analysis, biostatistical evaluation, and the interpretation of data was performed as described previously [7]. The samples were processed using KAPA HyperPlus Kit (Roche) according to the KAPA HyperCapture protocol (Roche), and custom hybridization probes (944 kbp of target sequence, including 765 kbp of coding regions of 300 genes; KAPA HyperChoice; Roche). The RNA samples were processed by amplicon RNA NGS using the Archer FusionPlex Sarcoma Expanded Kit (ArcherDX) according to the manufacturer’s protocol. The prepared libraries were sequenced by the NextSeq instrument (Illumina) using the NextSeq500/550 High Output Kit v2.5 (300 Cycles) according to the manufacturer’s protocol. Detailed pipelines of all NGS data analysis together with module settings are available upon request. The RNA NGS analysis revealed a fusion transcript of the *YAP1* (NM_006106: intron 3) to *TFE3* (NM_006521: exon 7; Fig. 2). DNA sequencing analyses revealed pathogenic or likely pathogenic variant of the *MUTYH* gene: NM_001128425.1:c.1187G > A, p.(Gly396Asp), missense, frequency of mutated allele 49.6% (coverage 1248x); rs36053993, ClinVar ID 5294. The tumor was microsatellite stable with low tumor mutation burden (TMB = 0 mut/Mb).

## Discussion

CCSTL is a rare tumor characterized by its morphological similarities with hemangioblastoma, but with a different IHC profile. Histologically, the tumor is usually composed of spindle shaped or ovoid cells arranged in sheets or cords, with a delicate stroma. The nuclei are usually monomorphic, round to oval, without atypia. Nevertheless, cases with more significant atypia have been describe. Mitoses are absent or rare. The cytoplasm of the tumor cells is pale eosinophilic or clear, but some cases showed only a minimal component of clear cells or no cytoplasmic clearing at all, so the term “clear cell tumor” has been questioned by some authors [2, 4]. An unusual feature in our case was the presence of occasional multinucleated giant cells, some with osteoclast-like features, which were non-neoplastic in origin. The presence of such cells is a known phenomenon also described in other tumors, including lung carcinoma [8].

Immunohistochemically, CCSTL is S-100 protein and inhibin negative, and generally lacks the IHC profile suggestive of any specific lineage of differentiation. Concerning the other examined markers, the tumors consistently expresses only vimentin. Our case showed diffuse expression of CD10 and IFITM1, but the significance of these markers for differential diagnosis is limited as only 1 from 4 previously examined cases showed expression of CD10 and expression of IFITM1 was not examined in any of the reported cases. IFITM1 does not seem to be a specific marker, as it is reported to be highly expressed in several mostly epithelial tumors such as colorectal, gastric, esophageal, and gallbladder carcinoma, in which it represents an independent prognostic biomarker [9]. However, in mesenchymal tumors its expression is less known and for diagnostic purposes, its main value lies in the diagnosis of endometriosis and endometrial stromal tumors [10, 11].

The differential diagnosis of CCSTL includes solitary fibrous tumor (SFT), sclerosing pneumocytoma and clear cell “sugar” tumor among the primary pulmonary neoplasms, from the secondary lung lesions namely metastasis of clear cell renal carcinoma, epithelial-myoepithelial carcinoma, and PEComa. The correct diagnosis should be achieved based on the combination of morphological and immunohistochemical features. From the immunohistochemical markers, especially TFE3 expression could be helpful. However, the TFE3 expression is not specific for this tumor, as it has previously been reported in other tumors of different histogenesis (including the MiT family translocation-associated renal cell carcinoma, perivascular epithelioid cell tumors, subset of epithelioid hemangioendotheliomas, and some others) [12–14]. In doubtful cases, molecular testing can be useful, as the characteristic feature of the CCSTL seems to be the *YAP1-TFE3* fusion, which was found in 9 of the 10 molecularly examined cases, as well as in our case. However, we should be aware that although the *YAP1-TFE3* gene fusion seems to be a defining and characteristic feature of CCSTL, this fusion has also been found in a subset of epitheloid hemangioendothelioma. Nevertheless, the IHC profile and morphology of epitheloid hemangioendothelioma is significantly different [14–17]. To date, the only other tumor type harboring the *YAP1-TFE3* fusion was single case of cutaneous low-grade fibromyxoid neoplasm [18].

In conclusion, we have described a case of CCSTL with the presence of the characteristic *YAP1-TFE3* fusion and have expanded the knowledge about the morphological features of this tumor by the presence of multinucleated giant cells and IHC expression of IFITM1. The immunohistochemical profile of these tumors is nonspecific, with the exception of TFE3 expression, which seems to be a common feature of these tumors. In doubtful cases, molecular testing focused on the *YAP1-TFE3* fusion should be performed. While originally these tumors were regarded as benign lesions, 3 tumors from the 18 cases reported to date behaved in a malignant fashion [2, 6]. In one of those the tumor was highly aggressive and the patient died 7 months after the diagnosis. Based on this data, the tumor should be regarded as an entity with malignant potential.

## Supplementary Information

**Additional file 1: Table 1. **List of antibodies.

## Data Availability

The datasets generated during the current study are available from the corresponding author on reasonable request.

## Abbreviations

CCSTL: Clear cell (hemangioblastoma-like) stromal tumor of the lung
IHC: Immunohistochemical
SFT: Solitary fibrous tumor
